# Acquisition of landmark, route, and survey knowledge in a wayfinding task: in stages or in parallel?

**DOI:** 10.1007/s00426-020-01384-3

**Published:** 2020-07-14

**Authors:** Kyungwan Kim, Otmar Bock

**Affiliations:** Institute of Exercise Training and Sport Informatics, German Sport University Cologne, Am Sportpark Müngersdorf 6, 50933 Cologne, Germany

## Abstract

According to an influential concept, humans acquire spatial knowledge about their environment in three distinct stages: landmark knowledge is acquired first, then route knowledge, and finally survey knowledge. The stage concept has been challenged by studies which observed that in a wayfinding paradigm, route, and survey knowledge emerge at the same time and; therefore, were seemingly acquired in parallel. However, this experimental evidence is not conclusive because the above studies suffered from a ceiling effect. The present study was designed to overcome the ceiling effect by increasing the complexity of the wayfinding task. We asked 60 young participants to find their way through an urban environment rendered in virtual reality, and assessed their landmark, route, and survey knowledge after each of ten trials. We found that all three types of knowledge gradually increased from the first to the last trial. We further found that correlations between the three types of knowledge increased from trial to trial. This outcome disagrees profoundly with the stage concept, but is compatible with the parallel concept. Specifically, it is in accordance with the view that landmark, route, and survey knowledge are acquired by multiple overlapping and interacting processes: those processes may start out more or less independently in the first trial but, due to common constraints or synergies, may gradually increase their cooperation during subsequent trials.

## Introduction

According to an influential concept (Siegel & White, 1975), humans acquire spatial knowledge about their environment in three successive stages. We first acquire *landmark knowledge*, i.e., we memorize the appearance of distinctive objects along the way. Once this is accomplished, we acquire *route knowledge*, i.e., we learn the sequence of direction choices at intersections. This sequence can be coded directly, e.g., as “left, then straight, then right” (Tlauka & Wilson, 1994), or it can be coded as landmark–direction associations, e.g., as “turn left at the gas station, then turn right at the bakery” (Kuipers, 1978). Once this is accomplished as well, we acquire *survey knowledge*, i.e., we form an internal representation or ‘cognitive map’ of the environment (Lynch, 1960; Rand, 1969; Siegel & White, 1975). Survey knowledge is independent of our own position, and enables us to find shortcuts, bypasses and even completely new routes to our goal (O´Keefe & Nadel, 1978; Tolman, 1948).

Indirect support for the stage concept comes from developmental studies: in childhood, the ability to use landmark knowledge emerges first, followed by route knowledge and then by survey knowledge (e.g., Hermer & Spelke, 1994; Jansen-Osmann & Wiedenbauer, 2004; Lew, Bremner, & Lefkovitch, 2000; Schmelter, Jansen-Osmann, & Heil, 2009; Tonucci & Rissotto, 2001). However, this ontogenetic perspective provides no persuasive support for the stage concept: abilities may develop in stages, but this does not necessarily imply that they are used by adults in stages. Indeed, neuroimaging studies recently reviewed by Chrastil (2013) suggest that the three types of knowledge can be broken down into smaller components, and that some of those components may be acquired in parallel rather than in stages. The review further concludes that in some cases, survey knowledge may be acquired before route knowledge has been consolidated, and it proposes a fourth type of knowledge, termed “graph knowledge”.

Experimental evidence for or against the stage concept can be gathered by different paradigms and the findings yielded by those paradigms are not necessarily identical. This has been well documented by a group of studies which compared the development of spatial knowledge when participants studied the map of a particular environment, and when they walked through that environment virtually or really (Taylor, Naylor, & Chechile, 1999; Thorndyke & Hayes-Roth, 1982; Zhang, Zjerdeva, & Ekstrom 2014). Different types of spatial knowledge evolved along somewhat different trajectories in the map condition as compared to the walking condition, which indicates that findings yielded by one paradigm may not generalize fully to another paradigm.

Several studies dealt with the acquisition of spatial knowledge in a wayfinding paradigm. Participants walked or were driven through an unknown neighborhood, or they moved through a virtual environment by means of a keyboard, mouse, or joystick. Two of those studies administered tests of spatial knowledge after each trial, and found near-perfect route knowledge as well as substantial survey knowledge already after the very first trial, with little increase of survey knowledge later on (Gärling, Böök, Lindberg, & Nilsson 1981; Ishikawa & Montello, 2006). Four other studies tested for survey knowledge only after route knowledge became near-perfect, which was the case after 3–5 trials; at that time, survey knowledge was already substantial (Iglói, Doeller, Berthoz, Rondi-Reig, & Burgess 2010; Jansen-Osmann & Fuchs, 2006; Jansen, Schmelter, & Heil, 2010; Weisberg, Schinazi, Newcombe, Shipley, & Epstein, 2013). These findings have been interpreted as evidence against the stage concept (Siegel & White, 1975), according to which survey knowledge cannot be acquired during the route knowledge stage, and as support for the parallel concept (Montello, 1998), according to which survey knowledge is acquired concurrently with route knowledge.

Although the above reasoning is plausible, it is not conclusive. Experimental data documenting that both route and survey knowledge were substantial already at the first time of testing does not necessarily indicate that both types of knowledge were acquired in parallel. Rather, it also is conceivable that knowledge was acquired in stages, and that all three stages were already passed through at the time of first testing because the wayfinding task was so easy. If so, above experimental data would reflect a ceiling effect rather than evidence for the parallel concept. Indeed, the wayfinding tasks in above studies included only 2–8 landmarks and only 3–13 decision points (i.e., intersections where participants had to decide which way to continue) which, in our experience, is easy to master.

We are aware of only one study that provides credible experimental evidence against the stage concept and for the parallel concept. It documented that landmarks are easier to remember when metric information about the connecting hallways is provided, which suggests that at least landmark and route knowledge are acquired in parallel (Buchner & Jansen-Osmann, 2008). The present study presents further experimental evidence for the parallel concept. We decided to replicate the above inconclusive research, but to avoid ceiling effects. Therefore, we conducted a series of pilot tests to ensure that our wayfinding task suffers neither from floor nor from ceiling effects. Those pilot tests gradually increased task difficulty by adding more and more decision points, until finally about ten trials were needed to acquire substantial landmark knowledge. This final version of the task was then used in our main experiment.

## Methods

### Participants

Sixty young adults (35 males and 25 females, 28.6 ± 4.6 years of age) participated in this study. They were recruited by written and by online postings, as well as by personal contacts on the campus of the German Sport University Cologne. All participants were without physical or cognitive deficits by self-report. Persons who wore eyeglasses or contact lenses in their daily life continued to wear them in the experiment. This study was approved by the Ethics Committee of the German Sport University Cologne. All participants signed an informed consent statement before testing began, and were not paid for their contribution to our study.

### Materials

**Fig. 1:**
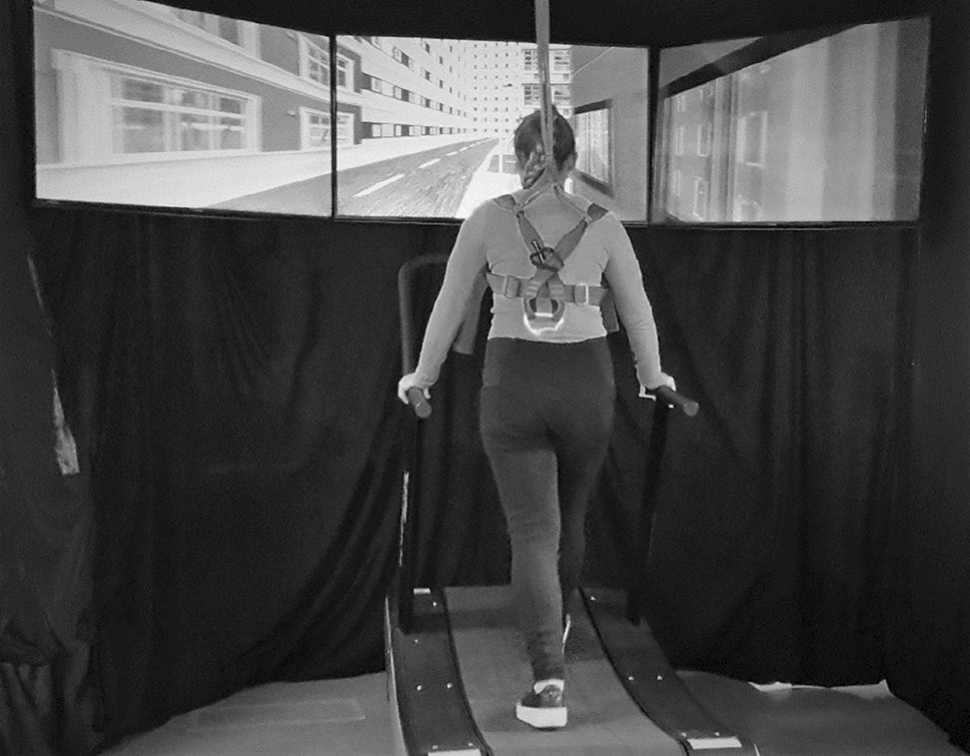
Experimental setup of VR city. A participant stands on the treadmilland faces VR City

Participants learned to find their way through a virtual reality city (VR City), developed by a commercial provider with the Unity™ game engine. A large-scale downtown area with numerous streets, buildings, props, and a community park were displayed on three integrated 46-in. TV screens, located at eye level (see Fig. 1). The middle screen was in line with the participants’ medio-sagittal plane, and the other two screens were located to the left and right at the angle of 106°.

**Fig. 2:**
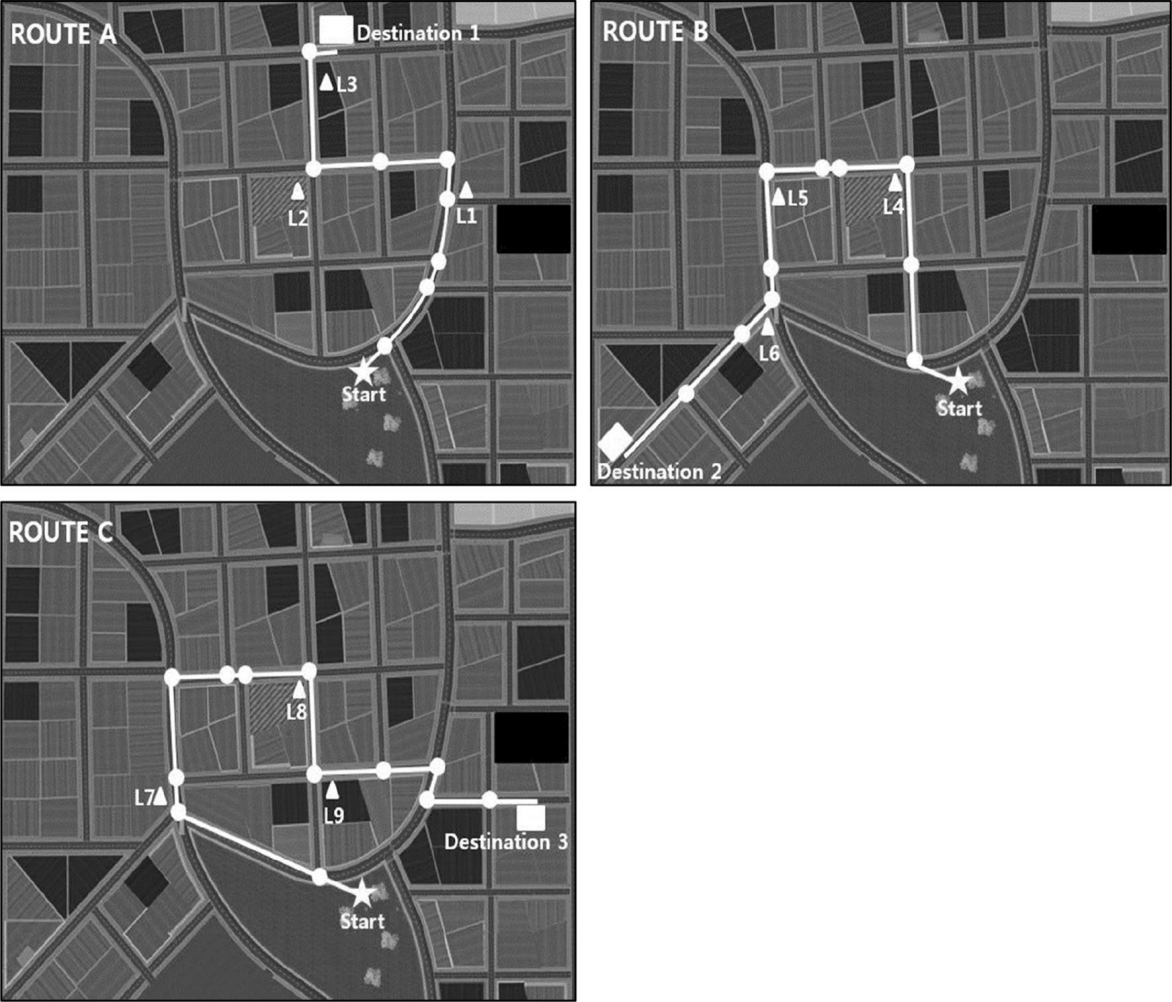
Map of VR-City. White lines represent the three routes that participants had to walk. All routes began at a common starting point, but each ended at a different destination. White circles indicate the location of 30 decision points, and triangles labelled by “Li” indicate the location of nine landmarks along the three routes

Participants progressed forward through this environment by walking at their self-determined speed on a non-motorized, gait-powered treadmill (Speedfit 1000, Vibrafit); to turn left or right, they pressed a switch attached to the left or right handrail, respectively, which led to a left- or rightward rotation of the virtual environment at a constant angular velocity. As a precaution against falls, participants wore a harness that was attached to the ceiling. Participants wore their daily clothes as they were in daily life. The middle monitor was in line with the participants’ medio-sagittal plane, and the other two monitors were positioned to the left and right at an angle of 106°, as if participants were actually moving in the VR City. Participants were then told that they will go along three different routes according to the experimenter’s instruction. Every route involved three landmarks (buildings, bus station or billboard). Route A involved 8 decision points, route B 10 and route C 12 decision points, for a total of 30 decision points (see Fig. 2).

### Procedure

An experimental trial consisted of a learning and a testing phase. During the learning phase, participants walked through VR City first along route A, then along route B, and finally along route C. Then, they stepped off the treadmill, and sat down for the testing phase, where they performed four computer-based tests of spatial knowledge. Participants completed ten trials, each consisting of a learning phase with three routes, and a testing phase with four spatial knowledge tests. Each learning phase took about five minutes and each testing phase about eight minutes, such that the total duration of the experiment was about (5 + 8) × 10 = 130 min.

Each learning phase began at a common starting point in the public park of VR City. Route A led participants from that point towards a bakery store. After reaching that destination, participants were teleported back to the starting point and subsequently walked along route B to a book shop. After reaching that destination, they were again teleported back to the starting point and walked along route C to a red house. The same three routes were used in the learning phase of all trials, i.e., participants experienced ten times the sequence route A—route B—route C.

The learning phase of the first two trials was experimenter-guided: as participants approached an intersection, they were told exactly which direction they should take. The learning phase of the remaining eight trials was self-guided: as participants approached an intersection, they called out which direction they planned to take, and the experimenter only corrected them if necessary. Thus, participants received instant error feedback and never took a wrong direction.

Each testing phase consisted of a recognition and a sequence test, both administered on a laptop PC, followed by a map and a direction test, both administered on a touchscreen tablet PC. The same four tests were administered in the testing phase of all trials.

#### Recognition test

**Fig. 3:**
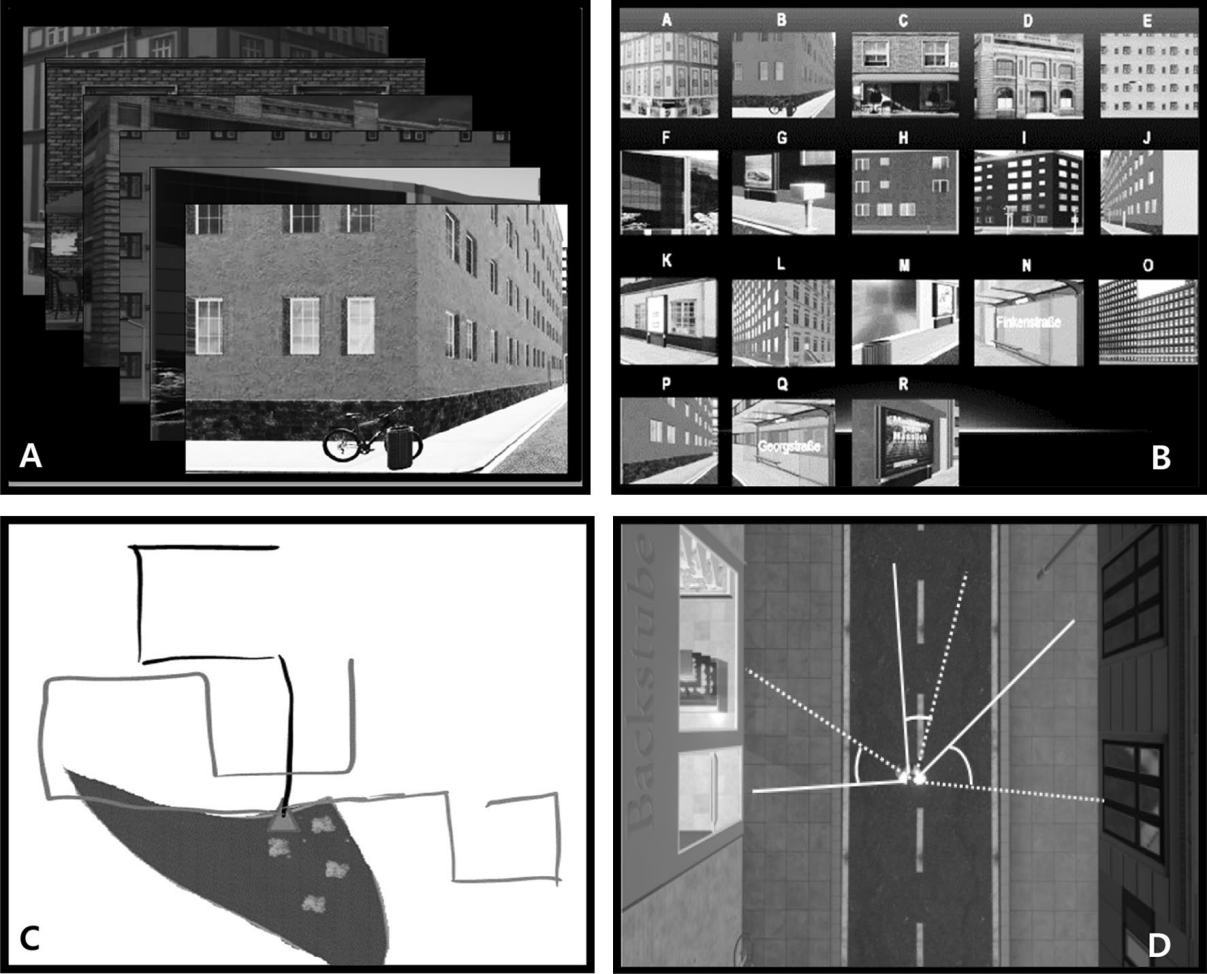
Screenshots of the four spatial knowledge tests. **a** *Recognition test*, 18 landmarks were presented sequentially, and participants had to indicate which ones they had encountered in VR City. **b** *Sequence test*, 18 landmarks were presented concurrently, and participants had to indicate the order in which they encountered nine of them. **c** *Map test,* the public park of VR City was presented in top view, along with the starting point of all routes (triangle); participants had to draw the three routes. **d** *Direction test*, one of the destinations was displayed, and participants had to draw a line to each of the order two destinations as well as to the starting point; this was then repeated for the other two destinations, and we calculated the absolute difference between drawn directions (dashed lines) and the pertinent correct directions (solid lines)

First-person views of eighteen intersections were displayed sequentially on a computer screen (Fig. 3a). Nine of them were the intersections with landmarks L1–L9, as encountered on each trial; they were depicted in the same perspective in which participants had just seen them. The other nine intersections were also from VR City, also had distinctive landmarks, but were not encountered on any trial; they were depicted in a comparable perspective. The intersections were presented in a mixed order for five seconds each, and participants estimated their familiarity on a 7-point Likert scale ranging from “fully unknown” to “fully known”. For each previously encountered intersection, participants earned one point when responding “fully unknown”, and seven points when responding “fully known”. For each previously non-encountered intersection, they earned 1 point when responding “fully known”, and seven points when responding “fully unknown”. Points were then added up, such that scores could range between 18 × 1 = 18 and 18 × 7 = 126 points. Random performance corresponded to a sum of (18 × 4 =) 72 points.

#### Sequence test

The same eighteen intersections were presented simultaneously on the monitor for 5 min (Fig. 3b). Participants were instructed to report, in proper order, the three intersections they encountered while walking on route A, then the three intersections from route B, and finally those from route C. One point was awarded for each landmark reported in the correct order on the correct route. Scores could; therefore, range between 0 and 9. Random performance corresponded to a score of three.

#### Map test

The public park of VR City was presented in top view on the touch screen, along with the common starting point of all three routes. Participants were asked to use a touch pen and draw on that touchable screen the three routes which they had just walked (Fig. 3c). They were reminded to draw all three turns on route A, all four turns on route B, and all six turns on route C. One point was awarded for each correct turn drawn along a given route in an uninterrupted sequence. For example, the correct sequence of turns on route A was “left, right, right”, and participants who drew this route as “left, left, right” therefore received only one point, because the sequence of correct turns was interrupted after the first turn. The points earned on all routes were then added up, and scores could; therefore, range between 0 and 13; random performance corresponded to a score of 2.78.

#### Direction test

The destination of the first route was displayed on the touch screen in top view. Participants were told to use a touch pen again and draw a line from the street median towards the destination of the second route. They were then asked to draw a line towards the destination of the third route, and finally to draw a line towards the starting point (Fig. 3d). The destination of the second route was then displayed, and line drawing was repeated. The destination of the third route was finally displayed, and line drawing was repeated once more. We calculated the absolute angular error between each line and the pertinent correct direction, and quantified drawing accuracy as the mean absolute error of all nine lines (three destinations × three directions). Scores could; therefore, range between 180° and 0°, and random performance corresponded to a score of 90°*.*

### Data analysis

We considered the recognition test to be a selective indicator of landmark knowledge. We; therefore, calculated landmark knowledge in percent as

1 $${\text{LK}}\;\left[ \% \right]\, = \,\left( {{\text{xr}}\,{-}\,{72}} \right)\, \times \,{1}00/{54}$$

where xr denotes the score yielded on the recognition test. Random performance on that test; therefore, corresponds to LK = 0%, and perfect performance to LK = 100%.

Similarly, we considered the direction test to be a selective indicator of survey knowledge. Therefore, we calculated survey knowledge in percent as:

2 $${\text{SK}}\;\left[ \% \right]\, = \,\left( {{9}0\,{-}\,{\text{xd}}} \right)\, \times \,{1}00/{9}0$$

where xd denotes the score yielded on the direction test. Random performance on that test; therefore, corresponds to SK = 0%, and perfect performance to SK = 100%. Note that the direction test does not distinguish between the different encoding strategies for survey knowledge that were recently described in literature (Zhong & Kozhevnikov, 2016).

The remaining two tests are not as selective, however. The sequence test is often regarded as a measure of route knowledge (e.g., Taillade et al., 2013), but it probably reflects landmark knowledge as well, since unknown landmarks are difficult to place in a sequence. The map test is often thought to be a measure of survey knowledge, but it has also been argued that it reflects route knowledge (e.g., Appleyard, 1970). We; therefore, decided to quantify route knowledge as common variance of the sequence test and the map test. To this end, data from both tests were submitted to principal component analysis, which yielded a single factor that explained 80.6% of total variance. Participants’ factor scores were taken as a measure of their route knowledge. Extracting the common variance from multiple variables is a standard procedure of latent variable analysis, see e.g., Loehlin and Beaujean (2016).

The onset of knowledge acquisition was quantified, separately for each type of knowledge, by fitting the knowledge scores with a set of models. Model 1 presumed that knowledge was acquired from the first trial on; to satisfy this model, scores were fitted by a single regression line. Model 2 presumed that knowledge was acquired from the second trial on; to satisfy this model, scores from trials 1 and 2 were fitted by a horizontal line *y* = *a* + 0 × *x*, where a was the mean score on trials 1 and 2; scores from trial 3 to 10 were fitted by a regression line. Models that presumed even later onsets of knowledge acquisition were calculated accordingly. For each model, goodness of fit was quantified as the root mean squared error (RMSE) between predicted and actual scores.

The association between different types of knowledge was determined by calculating the bivariate correlations between landmark and route knowledge, landmark and survey knowledge, as well as route and survey knowledge. This was done separately for each trial.

## Results

**Fig. 4:**
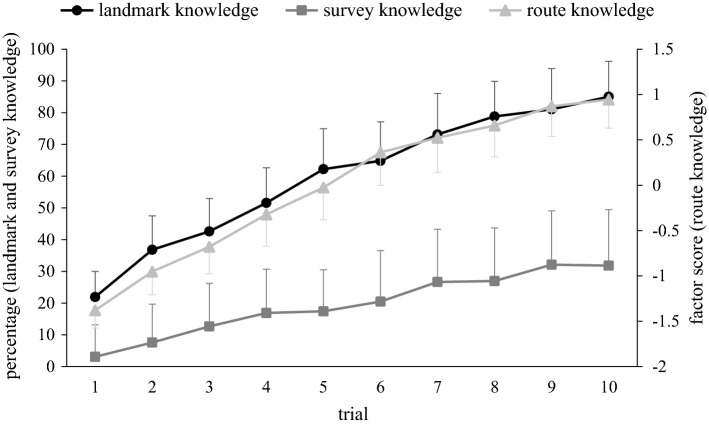
Developmental curves for landmark, route, and survey knowledge. Percentage scores of landmark and survey knowledge (left ordinate), plotted along with factor scores of route knowledge (right ordinate). Symbols represent across-participant means, and error bars are the pertinent between-participant standard errors. Note that the left and the right ordinate use different metrics (percentage versus factor scores), and curve slopes; therefore, cannot be compared

Figure 4 illustrates that all three types of knowledge increased continuously and concurrently from trial to trial. When compared with landmark knowledge, survey knowledge started lower on trial 1, and increased less throughout the subsequent trials.

Landmark knowledge scores were fitted by model 1 with RMSE = 22.924, which was lower than RMSE of all other models. Route knowledge scores were fitted by model 1 with RMSE = 0.6563 which was again lower than RMSE of all other models. Likewise, survey knowledge scores were fitted by model 1 with RMSE = 29.4707, which was once more lower than RMSE for all other models. We; therefore, found no evidence for the view that acquisition of any type of knowledge began later than on the very first trial.

**Fig. 5:**
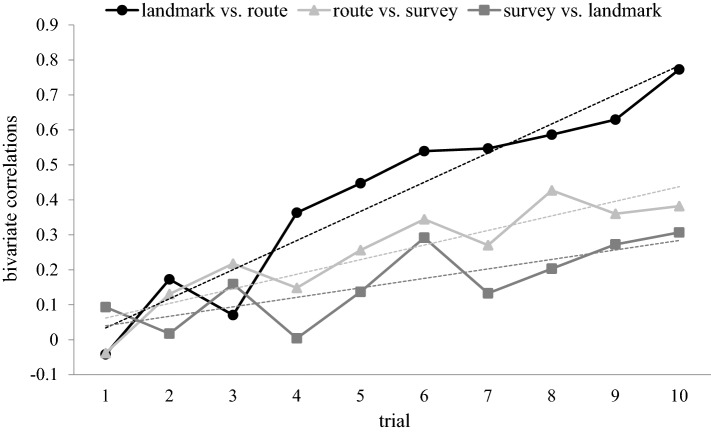
Developmental curves for bivariate correlations. Solid curves represent the correlations between landmark and route knowledge, landmark and survey knowledge, as well as route and survey knowledge, plotted separately for each trial. Broken lines are the regression lines for each knowledge pair. Note that correlations tended to increase from trial to trial, in particular those between landmark and route knowledge

Figure 5 shows that between-knowledge correlations tended to increase from trial to trial for all three knowledge pairs. Since the data from all three knowledge pairs met the prerequisites for linear regression (normality: all three Kolmogorov–Smirnov *d* < 0.167, all three *p* > 0.05; homoscedasticity: all three per inspection), we calculated a linear regression for each knowledge pair. The outcome without bootstrapping and with bootstrapping (*B* = 1000) was very similar; here we report the data with bootstrapping. *p* values are Bonferroni–Holm corrected for multiple testing. We thus yielded for the landmark—route pair a regression slope of 0.083 [95% CI 0.062, 0.102], which was significantly different from zero [*t* (28) = 9.334, *p* < 0.001]. For the route—survey pair, we yielded a slope of 0.042 [95% CI 0.026, 0.058], which again was significantly different from zero for [*t* (28) = 5.722, *p* < 0.001]. For the survey—landmark pair, we yielded a slope of 0.027 [95% CI 0.015, 0.041], which also was significantly different from zero [*t* (28) = 3.348, *p* < 0.05]. Thus, correlations increased significantly from trial to trial for all three knowledge pairs.

## Discussion

The present study deals with the acquisition of spatial knowledge in a wayfinding task that was complex enough to avoid ceiling effects. Tests of landmark, route, and survey knowledge were administered at the end of every trial, and revealed that landmark, route, and survey knowledge developed gradually and in parallel across all ten trials. Our findings should be interpreted with caution, however: they refer to the acquisition of spatial knowledge specifically in a wayfinding task, and may not fully extend to the acquisition of spatial knowledge in a map-studying task, or in other tasks (see “Introduction”).

By fitting different acquisition-onset models to our data, we documented that our findings are best compatible with the view that acquisition of spatial knowledge started from the first trial on; this was the case for all three types of knowledge. This outcome disagrees with the stage concept, which stipulates that only landmark knowledge is acquired during the initial trials, only route knowledge during subsequent trials, and only survey knowledge during yet later trials (see “Introduction”). Instead, our outcome is compatible with the parallel concept, which maintains that all three types of knowledge are acquired concurrently (see “Introduction”). This outcome confirms and extends the findings from an earlier study (Buchner & Jansen-Osmann, 2008). Thus, even if the ability to use spatial knowledge develops in stages throughout childhood (Cornell, Heth, & Broda, 1989; Sluzenski, Newcombe, & Satlow, 2004; Tonucci & Rissotto, 2001), acquisition of that knowledge during an actual wayfinding task in adults seems to proceed in parallel.

Parallel acquisition of landmark, route, and survey knowledge could be interpreted as evidence that all three types of knowledge are different manifestations of the same memory system, i.e., of generic spatial memory. Alternatively, parallel acquisition could be interpreted as evidence that the three types of knowledge rely on distinct memory systems, and that knowledge acquisition in all three systems was triggered by the same event, namely, by exposure to the first trial. However, both views are difficult to reconcile with the data in Fig. 5: a common memory system should yield high between-knowledge correlations in all trials, distinct memory systems should yield low between-knowledge correlations in all trials, but Fig. 5 shows a gradual increase in correlations from the first to the last trial. Such an increase fits better with the existence of three distinct, but cooperative memory systems: all three systems may start out independently, but then gradually increase their cooperation as the knowledge gained by one system helps to acquire knowledge by another memory system. Thus, for example, knowing the identity of landmarks could help to locate those landmarks along a route, and knowing the landmark positions, segment lengths, and decision alternatives along a route could help to form a survey representation of the environment. Alternatively, cooperation between memory systems could arise not because knowledge is shared, but rather because processing resources are shared, i.e., a given neuronal mechanism may contribute to the acquisition of different knowledge types. The latter view seems compatible with the hypothesis that spatial knowledge is acquired by multiple overlapping and interacting processes (Chrastil, 2013; Wolbers & Hegarty, 2010).

It is interesting to note that correlations between landmark and route knowledge increased from trial to trial at a higher rate than those between landmark and survey knowledge, and those between route and survey knowledge. In terms of the cooperative systems hypothesis, this finding suggests that the mutual cooperation between the memory systems for landmark and route knowledge is closer than their cooperation with the memory system for survey knowledge. It has indeed been proposed that the processing requirements for landmark and route knowledge are particularly similar (Chrastil, 2013).

It might be considered as a limitation of our study that the three routes were walked in a fixed order by all participants, rather than in a balanced order to avoid serial order effects. Note, however, that three routes that start at a common location are equivalent to a single route which repeatedly passes through a common location. Such looping routes are not unusual in real life, e.g., on an extensive, but not meticulously planned shopping trip. Our route design therefore reflects serial contingencies also found in natural wayfinding.

Another limitation of our study is that route knowledge was quantified as the common factor of two tests, rather than as the score on a single test. This was necessary because available wayfinding literature offers no agreed-upon test that is sensitive to route knowledge only.

In conclusion, our data provide evidence for the parallel concept of spatial knowledge acquisition, using a wayfinding task that is unhampered by ceiling effects. Furthermore, our data suggest that landmark, route, and survey knowledge may be acquired by multiple overlapping processes, rather than by three independent processes or by a single process.
