# Correction to: Acquisition of landmark, route, and survey knowledge in a wayfnding task: in stages or in parallel?

**DOI:** 10.1007/s00426-021-01567-6

**Published:** 2021-08-10

**Authors:** Kyungwan Kim, Otmar Bock

**Affiliations:** Institute of Exercise Training and Sport Informatics, German Sport University Cologne, Am Sportpark Müngersdorf 6, 50933 Cologne, Germany

## Correction to: Psychological Research (2021) 85:2098–2106 10.1007/s00426-020-01384-3

The article “Acquisition of landmark, route, and survey knowledge in a wayfnding task: in stages or in parallel?”, written by Kyungwan Kim and Otmar Bock., was originally published Online First without Open Access. After publication in volume 85, issue 5, page 2098–2106 the author decided to opt for Open Choice and to make the article an Open Access publication. Therefore, the copyright of the article has been changed to © The Author(s) 2020 and the article is forthwith distributed under the terms of the Creative Commons Attribution 4.0 International License, which permits use, sharing, adaptation, distribution and reproduction in any medium or format, as long as you give appropriate credit to the original author(s) and the source, provide a link to the Creative Commons licence, and indicate if changes were made. The images or other third party material in this article are included in the article’s Creative Commons licence, unless indicated otherwise in a credit line to the material. If material is not included in the article’s Creative Commons licence and your intended use is not permitted by statutory regulation or exceeds the permitted use, you will need to obtain permission directly from the copyright holder. To view a copy of this licence, visit http://creativecommons.org/licenses/by/4.0.

Open access funding enabled and organized by Projekt DEAL.

Original article has been corrected.

